# Changed Clonal Growth Form Induced by Sand Burial Facilitates the Acclimation of *Carex brevicuspis* to Competition

**DOI:** 10.1371/journal.pone.0121270

**Published:** 2015-03-30

**Authors:** Feng Li, Yonghong Xie, Lianlian Zhu, Li Jiang, Xinsheng Chen, Baihan Pan, Zhengmiao Deng

**Affiliations:** 1 Key Laboratory of Agro-ecological Processes in Subtropical Region, The Chinese Academy of Sciences, Hunan 410125, China; 2 Dongting Lake Station for Wetland Ecosystem Research, Institute of Subtropical Agriculture, Changsha, 410125, China; 3 University of the Chinese Academy of Sciences, Beijing 100049, China; Beijing Forestry University, CHINA

## Abstract

Both competition and burial are important factors that influence plant growth and structuring plant communities. Competition intensity may decline with increased burial stress. However, experimental evidence is scarce. The aim of this study was to elucidate the role of burial stress in influencing plant competition by investigating biomass accumulation, biomass allocation, and clonal growth performance of *Carex brevicuspis*, one of the dominant species in the Dongting Lake wetland in China. The experiment was conducted with two typical wetland species, *C*. *brevicuspis* (target plant) and *Polygonum hydropiper* (neighbor plant), in a target-neighbor design containing three densities (0, 199, and 398 neighbor plants m-2) and two burial depths (0 and 12 cm). The biomass accumulation of *C*. *brevicuspis* decreased with increment of *P*. *hydropiper* density in the 0 cm burial treatment. However, in the 12 cm burial treatment, biomass accumulation of *C*. *brevicuspis* did not change under medium and high *P*. *hydropiper* densities. The relative neighbor effect index (RNE) increased with enhancement of *P*. *hydropiper* density but decreased with increasing burial depth. The shoot mass fraction decreased with *P*. *hydropiper* density in the 12 cm burial treatments, but the root mass fraction was only affected by burial depth. However, the rhizome mass fraction increased with both *P*. *hydropiper* density and burial depth. The number of ramets decreased with increasing *P*. *hydropiper* density. With increasing burial depth and density, the proportion of spreading ramets increased from 34.23% to 80.44%, whereas that of clumping ramets decreased from 65.77% to 19.56%. Moreover, increased *P*. *hydropiper* density and burial depth led to greater spacer length. These data indicate that the competitive effect of *P*. *hydropiper* on *C*. *brevicuspis* was reduced by sand burial, which was reflected by different patterns of biomass accumulation and RNE at the two burial depth treatments. A change from a phalanx to a guerrilla growth form and spacer elongation induced by sand burial helped *C*. *brevicuspis* to acclimate to competition.

## Introduction

Interactions among species, including competitive, neutral, and positive relationships, play a major role in structuring plant communities [1, 2]. After decades of research, the importance of positive interactions in community organization has received increasing attention [3]. According to the stress-gradient hypothesis (SGH), the net negative competitive effects are more important under relatively benign environmental conditions, whereas positive facilitative effects are more important under harsher conditions [4–6]. This hypothesis has been documented in several studies [4, 6]. However, many other studies, including those conducted in the northwestern Negev desert of Israel and semi-arid Mediterranean steppes, have been failed to find support for this hypothesis [3, 7–9]. It seems that this divergence might account for differences in ecological factors (e.g., ecosystem types, stress intensities and stress types) and species traits [10]. Therefore, the generality of this hypothesis needs to be further tested [11–12]. To date, few studies have examined the relationship between competition and burial stress, which is a common stress in several types of wetlands, including coastal areas, river-connected lakes, and floodplains [13–14].

Sedimentation alters physical factors such as moisture, temperature, aeration, and other factors of the soil–plant microenvironment factors [15–16]. Plants have developed various adaptive characteristics to acclimate to heavy sedimentation, such as stem elongation, high-energy allocation to aboveground parts, high soluble sugar content, and photosynthetic efficiency [15, 17–18]. These adjustments allow plants to grow out of the sediment surface to escape an anaerobic environment [16].

Compared to non-clonal plants, clonal plants, the dominant wetland plant form, usually have more effective ways to acclimate to burial stress due to their different architectures such as branching angle, spacer length, and growth form [13, 17]. For instance, clonal plants can be classified into different clonal growth forms based on the spatial arrangement of ramets. The phalanx type and guerrilla type represent the endpoints in a continuum of possible growth forms [17, 19]. The guerrilla form, with spreading ramets, enables plants to spread quickly to escape unfavorable environments, while the phalanx form, with clumping ramets, allows plants to utilize locally abundant resources [17]. Studies have shown that the elongation of spacer length, plagiotropic growth, and an increased ratio of spreading ramets to clumping ramets are important ways for clonal plants to acclimate to sedimentation stress [13, 17, 20]. Moreover, these adjustments may also influence plant responses to other stresses such as competition. Resource availability is reduced under severe competitive situations, and clonal plants may respond to competitive conditions by positioning more ramets in competition-free patches, which can be obtained by spacer elongation. Therefore, it might be expected that spacer length elongation and an increasing proportion of spreading ramets induced by sand burial would also be favorable for the escape of plants from competitive conditions [19, 21]. However, experimental evidence is scarce.

The aim of this study was to test whether burial could reduce the strength of plant competition, and, if that is the case, whether this influence could be explained by the changes in clonal growth performance. In this study, young ramets of *Carex brevicuspis*, a typical wetland plant of Dongting Lake, were grown at two burial depths and three neighbor plant densities using a target-neighbor design. Here, *Polygonum hydropiper*, another dominant wetland clonal plant in Dongting Lake, was selected as a neighbor plant. Biomass accumulation, the relative neighbor effect index (RNE), biomass allocation, spacer length, number of ramets, and the proportions of clumping ramets and spreading ramets to total *C*. *brevicuspis* ramets were investigated to test the following hypotheses: (1) growth of *C*. *brevicuspis* will be inhibited by both higher burial depth and *P*. *hydropiper* density; (2) the influence of competition on plant growth will be reduced by burial according to the SGH; (3) the proportion of spreading ramets, spacer length, and rhizome mass fraction will increase with increasing burial depth and *P*. *hydropiper* density, and these will further increase under the combination of competition and burial stress.

## Materials and Methods

### Ethics statement

The collection of plant materials was approved by the East Dongting Lake National Nature Reserve. The two species used in the experiment are not endangered or protected species.

### Study area

Dongting Lake (111°40′–113°10′E, 28°30′–30°20′N) is the second largest freshwater lake and the most typical river-connected lake in China. The wetlands in this lake are characterized by large seasonal water level fluctuations, being completely flooded during May–October and dry during November–April. In this lake, wetland plants are usually distributed along a water-level gradient (e.g., high-elevation species include *Miscanthus sacchariflorus*, middle-elevation species include *C*. *brevicuspis* and *P*. *hydropiper*, and low-elevation species include *Phalaris arundinacea*). Sedimentation at a rate of approximately 3–7 cm year^−1^ has been confirmed as an important driving force regulating community succession in this lake [22–23].

### Plant materials

*C*. *brevicuspis*, a perennial rhizomatous sedge, is widely distributed in eastern mainland China and Taiwan [24]. The pseudoculm of the plant, made up of a series of overlapping leaf sheaths, is usually 20–55 cm in height. In Dongting Lake, *C*. *brevicuspis* usually forms mono-dominant communities or is co-dominant with other *Carex* species or *M*. *sacchariflorus* and *P*. *hydropiper*. Besides sexual reproduction, this species can produce two types of ramets, i.e. clumping ramets produced from buds of shortened rhizomes (usually <1 cm long) and spreading ramets from buds of long rhizomes (usually 2–25 cm long). Moreover, compared to sexual reproduction, asexual reproduction plays a more important role in vegetation recruitment [25]. *P*. *hydropiper* (Polygonaceae) is as an annual herb distributed widely around the world [18]. In Dongting Lake, *P*. *hydropiper* can overwinter through belowground rhizomes although the aboveground parts die [18]. In the *P*. *hydropiper* community, the most common concomitant species is *C*. *brevicuspis* and other species are less. These two species flower and fruit from April to May in Dongting Lake before flooding begins [17, 25]. The burial depth in the mixed community of *C*. *brevicuspis* and *P*. *hydropiper* was 0.4–3.1 cm in natural habitats based our field observations in 2009.

Plants (*C*. *brevicuspis* and *P*. *hydropiper*) were collected in March, 2014 from Junshan County (112°59′40.0″E, 29°22′17.7″N), East Dongting Lake. The vegetation was cut into small blocks (20 cm × 20 cm) for easy transport, and was then transported to an experimental field at the Dongting Lake Station for Wetland Ecosystem Research of the Chinese Academy of Sciences. The vegetation blocks were placed into plastic buckets, which contained 15 cm of soil (collected from a community of *C*. *brevicuspis* and that contained 1.45% organic matter, 4.04 μg g^-1^ exchangeable N, and 0.88 μg g^-1^ exchangeable P), to re-sprout new ramets. The plants were watered when necessary.

### Experimental set-up

**Fig 1:**
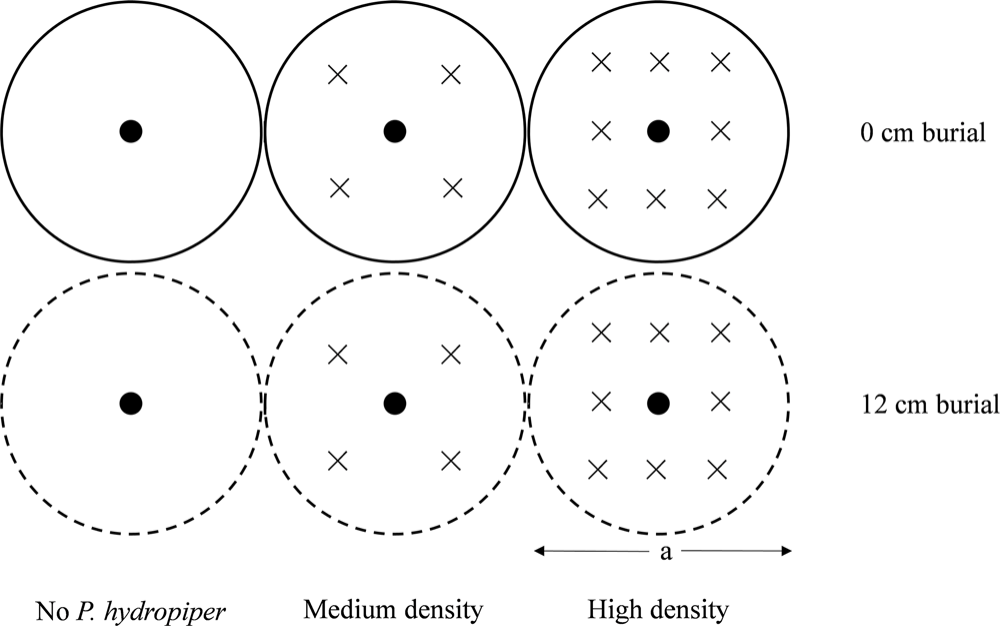
Diagram of the experiment design. “×” means neighbor plants and “●” means target plants. a = 16 cm.

The experiment combined two burial depths (0 and 12 cm) and three *P*. *hydropiper* densities (no plants: 0 plants m^-2^, medium: 199 plants m^-2^, and high: 398 plants m^-2^) in a factorial design with eight replicates, based on our field investigation (*P*. *hydropiper* densities ranged from 25 to 390 plants m^-2^). Therefore, the experiment included six treatments: two single-individual treatments (one *C*. *brevicuspis* plant per burial treatment) and four mixed-species treatments (one *C*. *brevicuspis* plant as a target plant, and 4 or 8 *P*. *hydropiper* plants as the neighboring plants per burial treatment; Fig 1). A total of 240 ramets (48 for *C*. *brevicuspis* and 192 for *P*. *hydropiper*) of similar size (3–4 leaves, about 20 cm in height for *C*. *brevicuspis* and 6–7 leaves, about 23 cm in height for *P*. *hydropiper*) were cut from plant cultures on March 20, 2014. They were planted into 48 pots (30 cm in height and 16 cm in diameter) filled with 15 cm of the same soil used for plant incubation.

Six pots (one per treatment) were placed into each of eight bigger plastic bins (98 cm × 76 cm × 68 cm), which were placed in an outdoor area with natural sunshine. A one-time burial treatment was initiated seven days after planting. The sand used for burial was collected from a local river (Xiang River) and contained 0.76 g kg^-1^ organic matter, 0.25 g kg^-1^ exchangeable N, and 0.06 g kg^-1^ exchangeable P. Tap water (containing 0.511 μg L^-1^ NH_4_^+^-N, 1.760 μg L^-1^ NO_3_^-^-N, and 0.527 μg L^-1^ PO_4_^3+^-P, pH = 7.2) was supplied when necessary. Holes were drilled in the bins at 15 cm depth to keep the water level at 0 cm and we also replenished the water once a week to prevent algal growth.

### Harvest

The plants were harvested five months after the experimental treatments. First, plant roots were carefully dug out by hand to keep the connection between ramets. Then, the plants were cleaned with tap water and the clumping ramets and spreading ramets were counted. Clumping ramets were defined as ramets produced from shortened rhizomes (spacer length < 1 cm), while spreading ramets were those produced from the distal part of long rhizomes (spacer length > 1 cm) [17]. The total number of ramets was the sum of the clumping ramets and spreading ramets. The proportion of spreading ramets and clumping ramets was determined as the ratio of the number of clumping ramets and spreading ramets to the total number of ramets, respectively. Spacer length was defined as the distance from each ramet to the original plant and it was measured with a 0.1 cm ruler. After these measurements, plants were divided into shoots, roots, and rhizomes, and were then oven dried at 80°C for 72 hours and weighed. Plant biomass was calculated as the sum of the mass of shoots, roots, and rhizomes. Shoot mass fraction, root mass fraction, and rhizome mass fraction were determined as the ratios of shoot mass, root mass, and rhizome mass, respectively, to total biomass.

### Relative neighbor effect index (RNE)

The intensity of interspecific competition was quantified using the relative neighbor effect index (RNE) [26]:

$$RNE=\left(P_{-N}-P_{+N}\right)/X$$ (1)

where P is the final biomass in the presence (+*N*) and absence (-*N*) of neighbors, and *X* is the larger value of *P*_-*N*_ or *P*_+*N*_. Biomass was In (*x*+1) transformed prior to statistical analysis to conform to the assumptions of normality and homogeneity of variance [27]. RNE is an improved version of the relative competitive intensity index [27]. The RNE enables both competitive and facilitative interactions to be quantified without bias on a scale of 1 to -1. A value of 0 indicates no interaction, a negative value indicates facilitation, and a positive value indicates competition.

### Statistical analysis

Two-way ANOVAs, with burial and *P*. *hydropiper* density as main factors, were performed to determine the main effects and interactions on biomass accumulation, RNE, biomass allocation, number of ramets, spacer length, and the proportion of spreading ramets and clumping ramets. Multiple comparisons of means were performed using Duncan’s test at the 0.05 significance level. Data were log_10_-transformed if necessary to reduce the heterogeneity of variances. Normality and homogeneity were tested using a Lilliefors test and Levene’s test, respectively. All analyses were performed using the software SPSS 15.0 for Windows.

## Results

### Biomass accumulation and RNE

**Fig 2:**
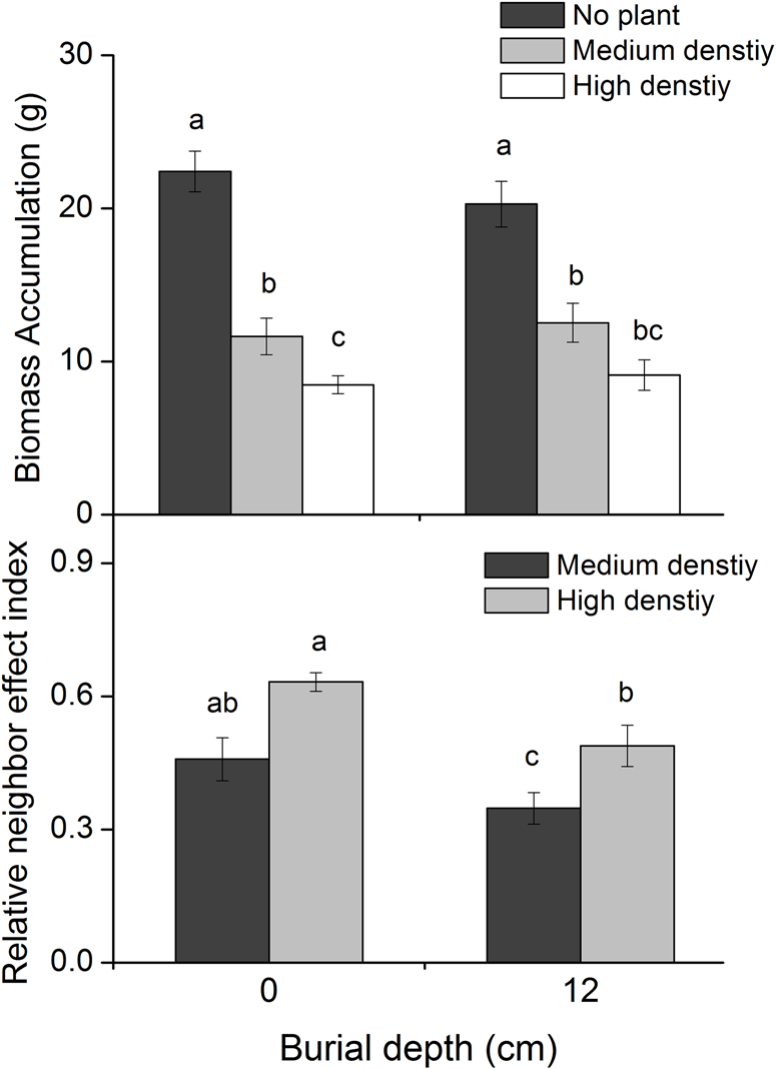
Biomass accumulation (mean ± SE, N = 8) and relative neighbor effect index (RNE; mean ± SE, N = 8) of *Carex brevicuspis* growing at two burial depths and three *Polygonum hydropiper* densities. Different letters indicate significant differences among treatments at the 0.05 significance level.

*P*. *hydropiper* density had a significant effect on *C*. *brevicuspis* biomass accumulation (Table 1; Fig 2), which was highest in the 0 cm burial + no plant treatment (22.4 ± 1.3 g per plant) and lowest in the 0 cm burial + high density treatment (8.5 ± 0.6 g per plant). In the same density treatment, biomass accumulation did not differ significantly between the two burial depths (Table 1; Fig 2). Biomass accumulation decreased significantly with increasing *P*. *hydropiper* density in the 0 cm burial treatments. However, in the 12 cm burial treatment, biomass accumulation exhibited similar patterns in the medium and high density treatments.

**Table 1:** Summary of two-way ANOVA analyses for biomass accumulation, RNE, biomass allocation, number of ramets, ramet proportions, and spacer length of *C*. *brevicuspis* under different competition and burial depth treatments (F-values).

Variable	Burial depth (B)	Competition (C)	B × C
Biomass accumulation	0.153^NS^	**25.767** ***	0.507^NS^
RNE	**10.680** *	**16.278** **	0.184^NS^
Shoot mass fraction	1.406^NS^	**4.115** *	1.241^NS^
Root mass fraction	**5.420** *	1.943^NS^	0.81^NS^
Rhizome mass fraction	**11.004** *	**5.690** *	2.339^NS^
Number of ramets	0.009^NS^	**10.448** ***	1.039^NS^
Proportion of phalanx ramets	**37.390** ***	**5.845** *	0.026^NS^
Proportion of guerrilla ramets	**37.390** ***	**5.845** *	0.026^NS^
Spacer length	**4.738** *	**46.070** ***	**4.207** *
d.f.	1	2	2

^NS^ *P* ≥ 0.05;

* *P* < 0.05;

** *P* < 0.01;

*** *P* < 0.001.

The RNE decreased significantly with increased burial depth and increased significantly with the enhancement of *P*. *hydropiper* density (Table 1; Fig 2). Compared to the 0 cm burial treatment, RNE decreased significantly in the 12 cm burial treatments (24.1% and 22.8% in the medium and high densities, respectively).

### Biomass allocation

**Fig 3:**
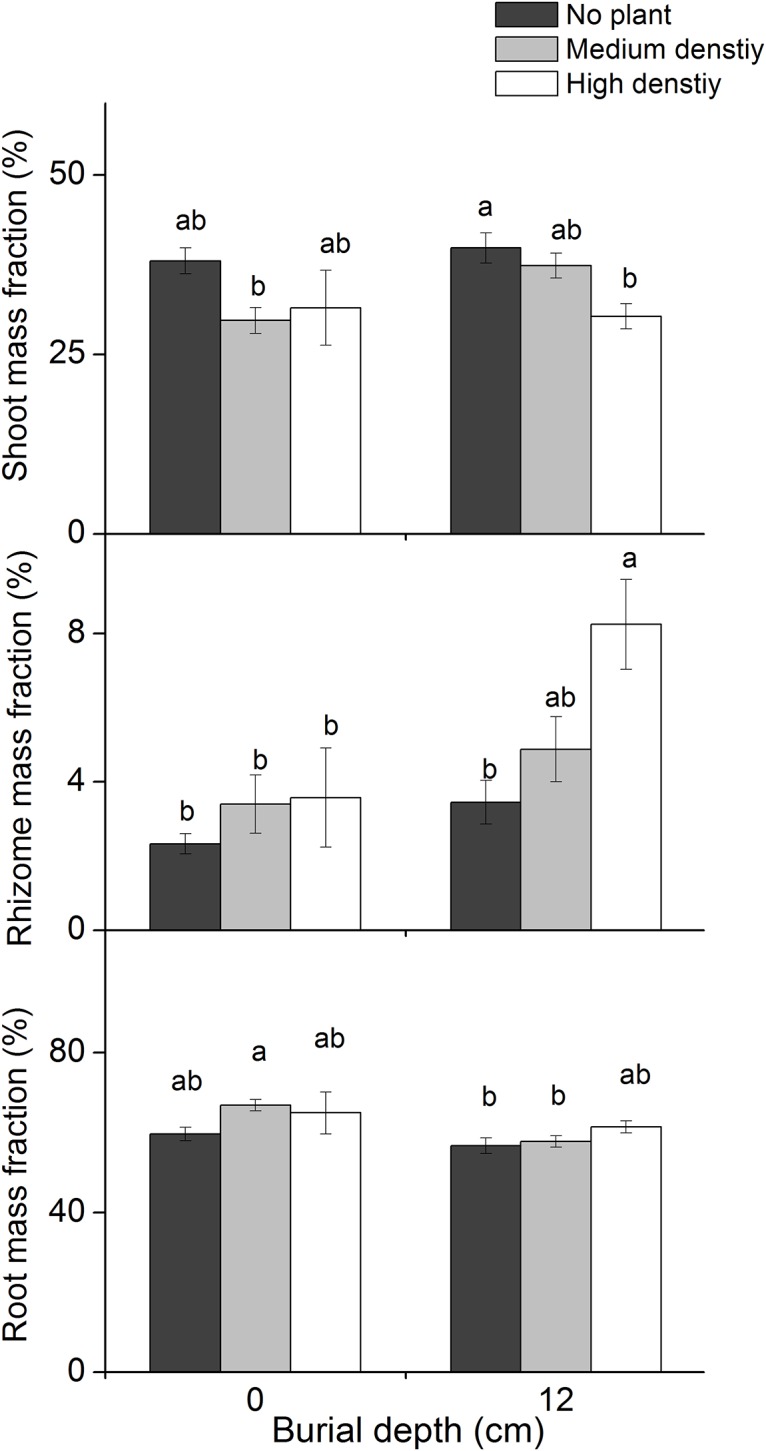
Biomass allocation pattern (mean ± SE, N = 8) of *Carex brevicuspis* growing at two burial depths and three *Polygonum hydropiper* densities. Different letters indicate significant differences among treatments at the 0.05 significance level.

The shoot mass fraction was significantly influenced by *P*. *hydropiper* density (Table 1; Fig 3), decreasing with increased *P*. *hydropiper* density in the 12 cm burial treatments (Fig 3). However, the root mass fraction was only significantly affected by burial depth (Table 1; Fig 3). In the medium density treatment, the root mass fraction decreased more in the 12 cm burial than in the 0 cm burial treatments (Fig 2). The rhizome mass fraction increased with increasing *P*. *hydropiper* density (Table 1; Fig 3) and burial depth (Table 1; Fig 3), which was highest in the 12 cm burial + high density treatment (8.2 ± 1.2%; Fig 3) and lowest in the 0 cm burial + no plant treatment (2.3 ± 0.3%; Fig 3).

### Number of ramets and spacer length

**Fig 4:**
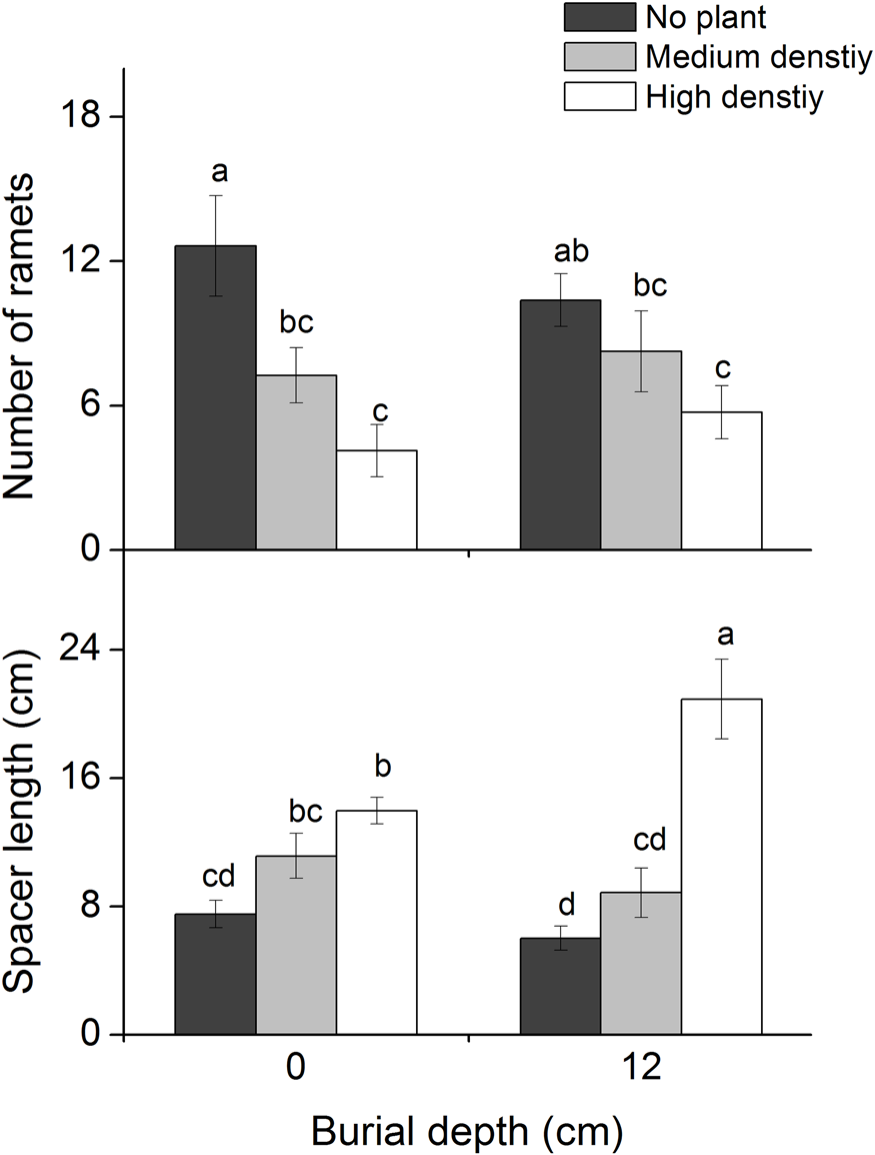
Number of ramets (mean ± SE, N = 8) and spacer lengths of *Carex brevicuspis* growing at two burial depths and three *Polygonum hydropiper* densities. Different letters indicate significant differences among treatments at the 0.05 significance level.

The number of ramets decreased with increasing *P*. *hydropiper* density (Table 1; Fig 4), but did not differ between the two burial treatments (Table 1; Fig 4). Spacer length increased significantly with increased *P*. *hydropiper* density (Table 1; Fig 4) and burial depth (Table 1; Fig 4). The highest spacer length occurred in the 12 cm burial + high density treatment (20.9 ± 2.5 cm), which was 3.48 times higher than the lowest value that occurred in the 12 cm burial + no plant treatment (6.0 ± 0.8 cm).

### Ramet proportions

**Fig 5:**
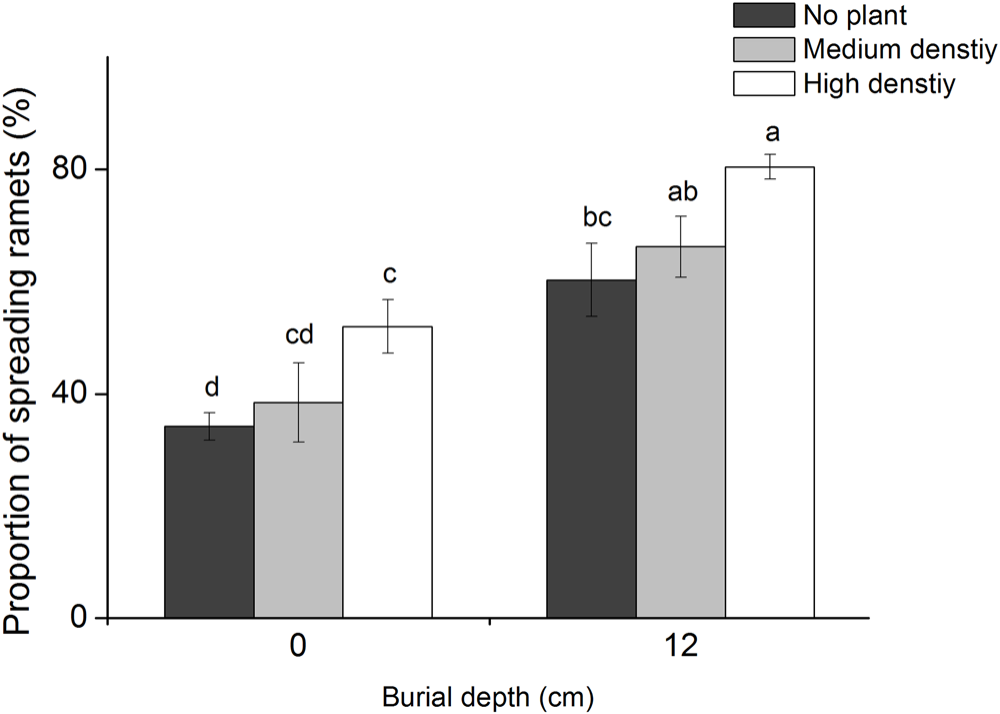
The proportion of spreading ramet out of the total number of ramets (mean ± SE, N = 8) in *Carex brevicuspis* growing at two burial depths and three *Polygonum hydropiper* densities. Different letters indicate significant differences among treatments at the 0.05 significance level.

The proportions of different ramet types were significantly influenced by both burial depth (Table 1; Fig 5) and *P*. *hydropiper* density (Table 1; Fig 5). For instance, the proportion of spreading ramets increased with burial depth and density, while that of clumping ramets decreased (Fig 5). The proportion of spreading ramets was highest in the 12 cm burial + high density treatment (80.4 ± 2.2%), and lowest in the 0 cm burial + no plant treatment (34.2 ± 2.5%). However, the proportion of clumping ramets displayed an opposite pattern.

## Discussion

The biomass accumulation and number of ramets of *C*. *brevicuspis* had insignificant changes between the two burial depths. These results are partly inconsistent with our hypothesis 1. Plants that grow in habitats with regular sand burial can usually tolerate a certain degree of burial, and some species even require regular burial to maintain high vigor [15]. Another study also found no significant influence of burial depth on the growth performance of *C*. *brevicuspis* [17]. The decrease in biomass accumulation and the total number of ramets among the different *P*. *hydropiper* density treatments suggested that a higher density of *P*. *hydropiper* intensified competition for resources and available growth space, then limited the growth of *C*. *brevicuspis*. This result was supported by the changing pattern of biomass accumulation in *P*. *hydropiper*, which was 1.31–1.38 times higher in the high density treatments than that in the medium density treatmtents. Moreover, the relationship between competition and neighbor plant density has been widely documented [6, 28].

The different biomass accumulation and RNE patterns of *C*. *brevicuspis* at the two burial depths indicated that the competition intensity of *P*. *hydropiper* to was reduced by burial, which was consistent with the SGH and hypothesis 2. A study conducted in Rookery Bay, Florida found that burial can cause net plant interactions to become more facilitative [29], which might account for the reduction in sand compaction caused by higher plant densities. Another explanation for these changing of plant interactions might be that higher *P*. *hydropiper* densities could alleviate soil anoxia by releasing oxygen into the soil through well-developed aerenchyma, which is supported by our other studies [16, 18]. Moreover, the influence of high density on increasing soil oxygen content has also been reported in other stress conditions [6, 30]. For instance, oxygen saturation under *Carex rostrata* was 2.1 times higher than that without vegetation treatment [30].

In our experiment, spacer length and the proportion of spreading ramets of *C*. *brevicuspis* increased significantly with burial depth and *P*. *hydropiper* density. These results are in agreement with hypothesis 3, and the results suggest that a trade-off existed between phalanx and guerrilla growth forms in *C*. *brevicuspis* in response to both burial and competition stress. Moreover, our results also indicate that spacer length was significantly affected by interactions between burial and plant density. The spacer length was significantly higher under 12 cm burial treatment than in the 0 cm burial treatment for the high *P*. *hydropiper* density treatment only, which indicated that the influence of burial changed significantly among different competitive intensities. This is particularly important considering that spacer elongation costs plants more energy. Regarding treatments with no *P*. *hydropiper* and medium *P*. *hydropiper* densities, the resource availability might be sufficient for plant growth owing to lower competition intensity. While in the high density *P*. *hydropiper* treatments, limited resource availability caused by high competition intensity might promote the placement of more ramets into favorable habitats through spacer elongation.

A change from the phalanx to guerrilla growth form has been widely confirmed as an effective way for plants to acclimate to various abiotic stresses, including burial [17], low nutrient availability [19], and shade [31]. However, plants have different ways to respond to competition [32]. For instance, stoloniferous *Prunella vulgaris* L. and *Glechoma hederacea* L. can increase stolon length in response to competition [33–34], while *Hieracium pilosella* L. and *Elymus lanceolatus* ssp. *lanceolatus* cannot [32, 35]. The difference might be correlated with plant resource levels. Plant can place ramets into favorable patches by increasing spacer length only when more resources are available from other parts of the genet [32]. Another determining factor might be the nutrient availability of unexploited patches. Plants are only capable of increasing stolon length in response to nutrient depletion by neighbors when the nutrient availability of unexploited patches is high enough [32].

In this experiment, the rhizome mass fraction increased with increasing burial depth and density, which corresponded with increased spacer length and the proportion of spreading ramets. These results support hypothesis 3. The production of long rhizomes involves greater energy costs. For instance, *Zostera noltii* can transform the nutrients from other organs to rhizomes to meet the energetic requirements of rhizome growth [36], and similar results have been reported in other studies [17, 37]. In the Dongting Lake wetlands, *C*. *brevicuspis* primarily adopts a phalanx growth strategy to monopolize locally abundant resources [38]. However, when treated with burial and varying levels of competition, *C*. *brevicuspis* can allocate more energy to produce a higher proportion of long rhizomes. This clonal growth change is an effective method for the avoidance of burial and competition stress for *C*. *brevicuspis*, and it might be an important reason why *C*. *brevicuspis* is a dominant species in the Dongting Lake wetland.
